# Influence of Light Regimes on Production of Beneficial Pigments and Nutrients by Microalgae for Functional Plant-Based Foods

**DOI:** 10.3390/foods14142500

**Published:** 2025-07-17

**Authors:** Xiang Huang, Feng Wang, Obaid Ur Rehman, Xinjuan Hu, Feifei Zhu, Renxia Wang, Ling Xu, Yi Cui, Shuhao Huo

**Affiliations:** 1 School of Food and Biological Engineering, Jiangsu University, Zhenjiang 212013, China; huangxiang@stmail.ujs.edu.cn (X.H.); fengwang@ujs.edu.cn (F.W.); obaid.sheikh@hotmail.com (O.U.R.); 1000005552@ujs.edu.cn (X.H.); lxu@ujs.edu.cn (L.X.); 2 School of Life Sciences, Jiangsu University, Zhenjiang 212013, China; feifzhu@ujs.edu.cn; 3 The First Geological Brigade of Hebei Provincial Bureau of Geological and Mineral Exploration and Development, Handan 056001, China; 15830051397@163.com

**Keywords:** microalgae, light, high-value products, artificial intelligence, plant-based foods

## Abstract

Microalgal biomass has emerged as a valuable and nutrient-rich source of novel plant-based foods of the future, with several demonstrated benefits. In addition to their green and health-promoting characteristics, these foods exhibit bioactive properties that contribute to a range of physiological benefits. Photoautotrophic microalgae are particularly important as a source of food products due to their ability to biosynthesize high-value compounds. Their photosynthetic efficiency and biosynthetic activity are directly influenced by light conditions. The primary goal of this study is to track the changes in the light requirements of various high-value microalgae species and use advanced systems to regulate these conditions. Artificial intelligence (AI) and machine learning (ML) models have emerged as pivotal tools for intelligent microalgal cultivation. This approach involves the continuous monitoring of microalgal growth, along with the real-time optimization of environmental factors and light conditions. By accumulating data through cultivation experiments and training AI models, the development of intelligent microalgae cell factories is becoming increasingly feasible. This review provides a concise overview of the regulatory mechanisms that govern microalgae growth in response to light conditions, explores the utilization of microalgae-based products in plant-based foods, and highlights the potential for future research on intelligent microalgae cultivation systems.

## 1. Introduction

In recent years, the plant-based (PB) food market has demonstrated substantial expansion. According to some government market reports, globally valued at approximately USD 43.77 billion in 2024, this market is projected to reach USD 85 billion by 2030, growing at a combined annual growth rate (CAGR) of 9–12%. Parallel to this trend, the plant-based milk sector exceeded USD 42 billion (CNY 300 billion) in 2022 and is expected to sustain a >20% annual growth rate post-2025. Notably, plant-based meat products accounted for 12% of fast food menu offerings in 2024, with the projections indicating this share will rise to 35% by 2030, while business-to-business (B2B) procurement volumes increase at 26% annually, largely driven by mounting consumer concerns regarding the environmental, health, and ethical implications of animal-based (AB) foods. Livestock production contributes significantly across the ecological, agronomic, socioeconomic, and economic domains, but the production of meat from pastured animals remains a major contributor to greenhouse gas emission, freshwater depletion, pollution, and biodiversity loss [1]. Despite the mounting interest in PB foods, most individuals still primarily consume AB products [2]. Consequently, PB food producers are compelled to formulate PB foods that resemble and taste like AB products, including meat, seafood, eggs, and dairy products [3,4]. The nutritional gaps in PB foods have driven the incorporation of functional components, such as natural pigments (e.g., carotenoids), plant-derived proteins (e.g., pea protein), and antioxidants (e.g., polyphenols), to enhance their nutritional adequacy [5]. Microalgal biomass is regarded as a significant renewable resource for extracting various active ingredients that possess antimicrobial or antioxidant properties and are utilized to enhance the properties of food products [6,7]. As a representative example, consider ‘blue foods’, aquatic-derived food resources that have been extensively historically consumed with a high nutritional value. Among these, specific microalgae species are particularly noteworthy due to their superior protein content, comprehensive essential amino acid profiles, widespread bioavailability, and low-level allergenicity [8]. Microalgae are widely recognized as a promising resource for the development of PB food products [5], including PB meat [9], PB seafood substitutes [10], dairy products [11], and flavoring agents for PB seafood [12]. The utilization of microalgae in many cultures for the production of value-added bioproducts has attracted considerable research interest in recent years, driven by the global pursuit of sustainability [13,14]. In addition to the direct use of algae as food additives [15], the utilization of microalgae allows for the production of high-value products, including proteins, carotenoids such as astaxanthin, and polyunsaturated fatty acids (PUFAs) [16]. The principal sources of these substances are plants and animals in nature [17,18]. A growing supply–demand imbalance exists due to the escalating industrial demand and the use of unsustainable resources. To meet the market demand for these products, identifying alternative sources is of the utmost importance. Microalgae represent a promising green resource, so the selection and large-scale cultivation of high-yield algal species will be a key area of future development [19]. To ensure commercial viability, optimal cultivation conditions and techniques must be established. For photoautotrophic microalgae, light is a critical factor influencing cellular growth and biosynthetic rates. Some examples of microalgae mass cultures using natural or artificial light sources from some of the world’s leading algae companies are listed in Table 1. Thus, investigating light conditions and advancing lighting technologies are essential for enhancing productivity. In current microalgal cultivation practices, the application of multiple abiotic stresses, such as modulated light intensity, specific light wavelengths, and controlled nitrogen source concentrations, has demonstrated its potential to enhance the production of commercially valuable bioproducts [20]. This strategy could enhance industrial scalability by improving process efficiency and product yield compared to those of the traditional cultivation methods.

To obtain large-scale microalgae cultures, it is imperative to exercise careful control over light conditions. This encompasses the intensity and spectral quality of light, which are instrumental in determining the culture’s success [21]. Among these parameters, light intensity and wavelength constitute the most critical environmental regulators, directly governing photosynthetic efficiency and growth dynamics in photosynthetic microalgae [22,23]. Microalgae generally employ photosynthesis to utilize outdoor sunlight or ordinary white light provided by an artificial light source [24]. However, photosynthetic pigments selectively absorb specific wavelengths of light. The use of red or blue light sources alone for microalgae cultivation has been shown to increase the biomass productivity of various microalgae compared to that of ordinary white light [25]. The dynamic adjustment of light intensity and quality is necessary to enhance the light utilization efficiency of microalgal cells in actual production. The advent of artificial intelligence (AI) technology has precipitated a paradigm shift in various industries, with the integration of AI becoming a prevailing trend in future development. This efficacy is attributable to the fact that machine learning algorithm (MLA) approaches furnish more comprehensive insights into the uncertainty of biological processes compared to the traditional phenomenological or dynamical models [26]. Numerous studies have been conducted on the utilization of AI for the continuous monitoring and real-time dynamic adjustment of microalgae culture processes. Recent research focuses on areas such as microalgae-based wastewater treatment [27], intelligent autotrophic cultures [28], and product intelligent extraction [29], among others. AI-based monitoring and dynamic light regulation are expected to play a pivotal role in future microalgal cultivation systems. The objective of this review is to examine the impact of light conditions on the growth and synthesis of valuable microalgae products, including proteins, oils, and natural pigments that can be utilized in the production of PB food products. It is important in the formulation of new PB foods. The insights derived from this review will contribute to the optimization of microalgae cultivation methods, thereby facilitating the acceleration of biomass accumulation and the biosynthesis of high-value products.

## 2. Methodology

**Figure 1:**
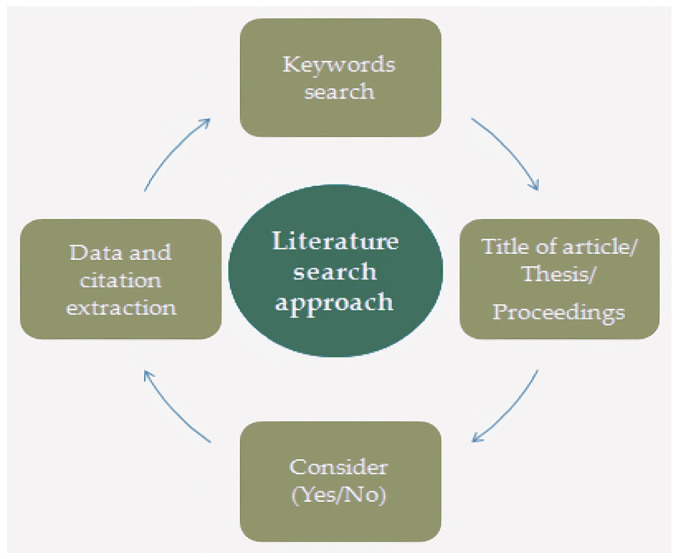
Literature search approach.

A comprehensive search strategy was implemented to identify relevant literature across three domains: (1) the light-mediated regulation of microalgal growth and biosynthesis, (2) microalgae applications in plant-based foods, and (3) the AI-driven optimization of microalgal cultivation and harvesting. A systematic literature assessment was therefore conducted to evaluate state-of-the-art knowledge. As shown in Figure 1, the search strategy employed core keywords, including ‘light parameters’, ‘microalgal cultivation’, ‘plant-based foods’, ‘microalgal biomolecules’ (encompassing proteins, fatty acids, and natural pigments), and ‘artificial intelligence’. To ensure efficient literature synthesis, core studies were systematically retrieved from multidisciplinary databases, including Web of Science, Scopus, PubMed, IEEE Xplore, ScienceDirect, SpringerLink, Wiley Online Library, Taylor & Francis Online, MDPI platforms, AGRO, Hindawi, Google Scholar, and China National Knowledge Infrastructure (CNKI). Following initial keyword searches, the identified literature underwent systematic assessment against predefined eligibility criteria for inclusion/exclusion. Articles directly aligning with the core search keywords were prioritized for retention. The metadata (titles, keywords, abstracts, authors, and references) extracted from selected publications constituted the primary dataset. Non-English publications were excluded. Ultimately, the relevant manuscript sections of 171 articles meeting all the criteria were thematically synthesized.

## 3. Effects of Light Changes on Photosynthesis and Growth of Microalgae

### 3.1. Microalgal Photoadaptation and Biomass Accumulation Under Variable Light Intensities

**Figure 2:**
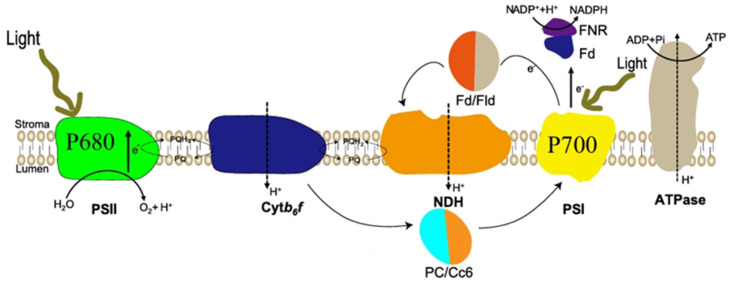
Photosynthetic system of microalgae cells, PSI: photosystem I; PSII: photosystem II; FD: Ferredoxin; FNR: Fd-NADP reductase; Fld: Flavodoxin; NDH: NAD(P)H dehydrogenase complex; PC: plastocyanin; Cc6: cytochrome; Cytb6/f: plastocyanin. NDH: NAD(P)H dehydrogenase complex; PC: plastocyanin; Cc6: cytochrome; Cyt b6/f: cytochrome b6-f complex; PQ: plastoquinone.

The photosynthetic pigments present in eukaryotic algal cells form a complete vesicle network with a vesicle-like membrane; the vesicles can be stacked or penetrate through the stroma [30] (Figure 2). Changes in light have been shown to primarily alter algal cell growth by affecting electron flow and reducing power [31]. Photosynthesis is one of the most sensitive physiological processes in microalgae, undergoing substantial structural and functional changes in the photosynthetic apparatus under diverse environmental conditions. These responses include modifications in light-harvesting complexes, antenna systems, and reaction centers, along with adjustments in excitation energy distribution between photosystem I (PSI) and photosystem II (PSII) [32]. Within an optimal range, increasing light intensity enhances electron flow through both PSI and PSII, thereby promoting the generation of ATP and NADPH. However, under excessive light conditions, the photosynthetic electron transport chain may become saturated, leading to its over-reduction and potential photodamage. To mitigate this, alternative dissipation mechanisms, such as non-photochemical quenching and cyclic electron flow, are activated to safely dissipate excess excitation energy and protect the photosynthetic machinery [33]. Carotenoids located within the photosynthetic reaction center P680 play a crucial role in protecting against photooxidative damage.

Photosynthetically active radiation (PAR) refers to the portion of solar radiation that can be utilized by photosynthetic organisms, typically defined within the 400–700 nm wavelength range. Although chlorophylls absorb light primarily in the blue and red regions of this spectrum, their absorption capacity is limited to specific wavelengths. To broaden the usable spectrum, photosynthetic organisms employ light-harvesting complexes (LHCs), which contain numerous chlorophyll molecules and carotenoids that collectively capture and transfer excitation energy to the reaction centers [34,35]. In addition to their structural role within the photosynthetic apparatus, carotenoids act as auxiliary light-harvesting pigments and photoprotective agents, quenching reactive oxygen species (ROS) and dissipating excess excitation energy, thereby reducing photodamage and photoinhibition in photosynthetic organisms exposed to light stress [36]. The intensity of incident light plays a critical role in regulating photosynthetic activity, which, in turn, governs the growth dynamics and biomass productivity of microalgae. Variations in light intensity result in differential growth rates and accumulation patterns across microalgal species. Additionally, the contents of cytochromes and lipids, as well as the composition and ratio of fatty acids, will also differ based on these light conditions [37]. To ensure the stable production of PB-food additives from microalgae, it is necessary to provide suitable light conditions for photosynthetic algae. Ensuring an adequate supply of light radiation is a major challenge in photoautotrophic microalgae cultivation, as it is essential to provide the appropriate amount of light to prevent both light limitation and photoinhibition during growth [37]. To ascertain the optimal light intensity for the cultivation of high-value microalgae, the impact of a diverse range of light intensities on the growth and biosynthesis of various microalgal species has been studied [38,39,40]. In most experiments on well-studied high-value microalgae, the light intensities ranged from 50 to 1500 μmol photons m^−2^ s^−1^, with optimal growth typically occurring from around 60 to 200 μmol photons m^−2^ s^−1^ (Table 2).

The growth rate of the diatomaceous microalgae *Phaeodactylum tricornutum* was found to be approximately 2 × 10^6^ cells ml^−1^ day^−1^ at an initial light intensity of 20 μmol photons m^−2^ s^−1^. However, when the light intensity was increased to 200 μmol photons m^−2^ s^−1^, the growth and division rate increased threefold [55]. The impact of light on photosynthesis is significant. Upon adjusting the light intensity from low (40 μmol photons m^−2^ s^−1^) to high (350 μmol photons m^−2^ s^−1^), it was observed that photosynthesis and respiration increased, and plastid–mitochondria contact was boosted [56]. The rate of carbon fixation by cellular photosynthesis was increased, resulting in the increased accumulation of organic matter within the cells. The cellular metabolism and biosynthesis of *P. tricornutum* were found to be significantly increased. Nevertheless, when light intensity exceeded 500 μmol photons m^−2^ s^−1^, the growth rate ceased to increase in tandem with the intensification of illumination. Instead, a decline in growth was observed, accompanied by the emergence of a negative effect on growth stagnation. High-intensity light impedes the process of cellular photosynthesis, reducing the rate of the synthesis of photosynthetic pigments [57].

In an outdoor bioreactor, Metsoviti et al. [58] observed the notable impact of light intensity on the growth and biomass productivity of *Chlorella vulgaris*. Terrestrial photosynthetically active radiation (PAR) intensities exhibit substantial variability depending on geographic location, seasonal changes, and atmospheric conditions. Increased light availability during sunnier periods has been shown to promote *C. vulgaris* growth, particularly by enhancing specific growth rates, the maximum optical density, and the overall biomass productivity. Concurrent studies by Khoeyi and Seyfabadi yielded analogous results, observing a 20% surge in biomass productivity upon elevating the light intensity to which *C. vulgaris* was exposed from 65 μmol photons m^−2^ s^−1^ to 78 μmol photons m^−2^ s^−1^ [59]. *C. vulgaris* is relatively less sensitive to changes in light intensity and can maintain good growth under higher-intensity light. Some studies have also shown that *C. vulgaris* can grow under extremely intense light. Other researchers have successfully cultivated *C. vulgaris* at ultra-high light intensities [60] (7000 μmol photons m^−2^ s^−1^) using a strobe effect with flickering light [61] rather than continuous exposure. Additionally, the variations in light intensity also significantly influenced the photosynthetic pigment content of *C. vulgaris*, affecting both cell growth and division rates. The concentration of chlorophyll decreased by more than 20% with increasing light intensity, whereas the content of *β*-carotene exhibited a contrasting trend, increasing by approximately 30% with increasing light intensity [62]. The primary rationale for this phenomenon is that excessive light intensity can result in light saturation, which may potentially lead to cellular damage and the disruption of photosynthetic processes. Carotenoids serve as vital antioxidants that mitigate the adverse effects of light saturation, including photoinhibition, on biological systems [63]. The researchers delineated the boundaries between weak and strong light by observing alterations in the growth pattern of *Haematococcus pluvialis* cells and the photoinhibition point [64]. Based on the experimental results, 100 μmol photons m^−2^ s^−1^ was selected as the optimum light intensity for *H. pluvialis*. At this time, the biomass of *H. pluvialis* was 4 g/L, and the biomass productivity value was 80 mg L^−1^ d^−1^. The aforementioned high-value microalgae exhibit sensitivity to fluctuations in light intensity. Maintaining photosynthetic efficiency in microalgal cultures requires adaptive light regulation. This can be achieved through feedback control systems that continuously monitor cell density and dynamically adjust light intensity to maintain irradiance within species-specific optimal ranges.

### 3.2. Microalgal Photoadaptation and Biomass Accumulation Under Variable Light Quality

The metabolic processes of microalgae cells can be influenced by light of a specific spectral composition, resulting in the accumulation of proteins, carbohydrates, or lipids [65]. Consequently, different wavelengths of light have been demonstrated to alter the growth of algal cells and the synthesis of substances. Jungandreas et al. [66] examined the impact of red light (RL), blue light (BL), and natural white light (WL) on the growth rate of *P. tricornutum*. The findings indicated that red light could facilitate the growth of *P. tricornutum* during the initial phase of cultivation; however, in the subsequent phase, the impacts of the three light qualities on the density of *P. tricornutum* cells were nearly indistinguishable. Significant differences in primary photosynthetic activity were observed between RL- and BL-acclimated microalgal cells. Specifically, the quantum yield of PSII across a range of light intensities was markedly lower in the BL-acclimated cells, indicating reduced photochemical efficiency under BL conditions. The synthesis of photosynthetic pigments in *P. tricornutum* was markedly influenced by different light qualities. Both RL and BL were observed to promote photosynthetic pigment synthesis, with RL exhibiting a more pronounced effect than BL [66]. In contrast to the findings observed in the case of *P. tricornutum*, the study demonstrated that the utilization of BL as a light source resulted in the enhancement of the optimal light intensity required for the growth of *C. vulgaris* [67]. The shorter wavelength of BL activates specific photoreceptor proteins in *C. vulgaris*, thereby promoting *C. vulgaris* biomass accumulation. The concentrations of chlorophyll a, chlorophyll b, and *β*-carotene increased under BL conditions at the same light intensity [24]. In contrast to the positive effect of BL on the growth of *C. vulgaris* cells, RL has been shown to cause damage to these cells and to reduce their growth rate [68]. Both red and blue light have been demonstrated to promote the growth and reproduction of *H. pluvialis*, and both of these variables accumulate greater biomasses in comparison to WL. Furthermore, the astaxanthin concentration of *H. pluvialis* has been shown to increase significantly in response to BL, reaching levels that are eight times higher than those observed under WL and two times higher than those observed under RL [69]. As demonstrated in the relevant literature, reactive oxygen species (ROS) have been shown to function as a cellular signal, thereby activating cellular metabolism in *H. pluvialis* [70], thereby promoting astaxanthin synthesis. BL undoubtedly promotes astaxanthin synthesis by stimulating the metabolism of *H. pluvialis*, leading to the production of ROS. Consequently, it is imperative to adjust the light quality according to the microalgae species during the actual cultivation process. The utilization of a combination of light-emitting diode (LED) lights of different colors as a light source has been demonstrated to be an effective solution to this problem.

## 4. Light Conditions for the High-Value Products of Microalgae Synthesis

The extraction of microalgae biomass and the subsequent analysis of the proteins, fatty acids, natural pigments, polysaccharides, and other substances present within this biomass are of significant importance in the realm of raw materials and food additives for PB foods (Table 3). Therefore, it is necessary to clarify the effects of changing light conditions on the synthesis and accumulation of these important nutrients in the algal cell. Microalgae productivity was found to be increased by regulating light factors (Table 4).

### 4.1. Long-Chain Unsaturated Fatty Acids

**Figure 3:**
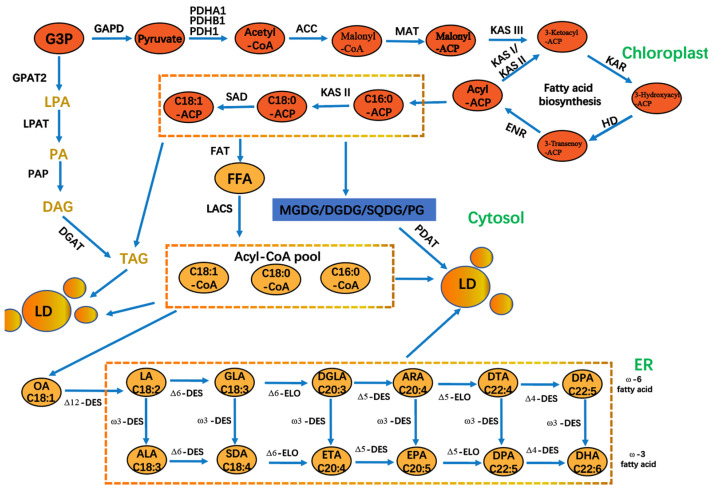
The process of PUFA biosynthesis in *P. tricornutum* cells. GAPD: glyceraldehyde 3-phosphate dehydrogenase; PDH: pyruvate dehydrogenase; ACC: acetyl-CoA carboxylase; MAT: malonyl-CoA-acyl carrier protein transacylase; KAS: 3-ketoacyl-ACP synthase; HD: 3-hydroxyacyl-ACP dehydratase; ENR: enoyl-ACP reductase; KAR: *β*-ketoacyl carrier protein reductase; ADS: acyl-CoA desaturase; DGAT: acyl-CoA: diacylglycerol acyl transferase; FAT: fatty acyl-ACP thioesterase; FFA: free fatty acid; DGDG. DGDG: digalactosyldiacylglycerol; MGDG: monogalactosyldiacylglycerol; PG: phosphatidylglycerol; SQDG: sulfoquinovosyldiacylglycerol; LD: lipid droplets; PDAT: phosphatidylglycerol; SQDG: sulfoquinovosyldiacylglycerol droplets; PDAT: phospholipid diacylglycerol acyltransferase; G3P: glycerol 3 phosphate; GPAT: acyl-CoA glycerol-3-phosphate acyl transferase. LPAT: acyl-CoA lysophosphatidic acid acyl transferase; PAP: phosphatidic acid phosphatase; LACS: long chain acyl-CoA synthetase; PDH: pyruvate dehydrogenase. PDH: pyruvate dehydrogenase.

A plethora of studies demonstrated the favorable effects of PUFAs on human health [81]. These include the ability to regulate blood pressure, strengthen the immune system, and reduce inflammation [82]. The addition of PUFAs to PB protein foods can increase their nutritional value, improve their water-binding capacity, and enhance their fat-binding properties [83]. Microalgae fatty acids can be a high-quality source of PUFAs in plant-based foods. The synthesis of PUFAs requires desaturases and elongases, which facilitate the formation of double bonds and carbon chain extension in fatty acids [84]. This process is accompanied by a significant energy expenditure, ultimately leading to the production of PUFAs (Figure 3) [85,86]. *Phaeodactylum tricornutum*, an important marine diatom, has emerged as a promising candidate for industrial biotechnology in recent years. Its rapid growth, robust metabolite production, and amenability to genetic engineering have established it as a model organism in algal bioengineering [87]. The market demand for *Phaeodactylum tricornutum* is expected to increase in parallel with the growing consumer interest in natural and organic ingredients. This trend is further driven by the rising demand for high-value bioactive compounds, such as eicosapentaenoic acid (EPA), fucoxanthin, and exopolysaccharides, for use in functional foods, nutraceuticals, and cosmetics [88]. *P. tricornutum* is regarded as a novel marine resource due to its abundance of EPA [89]. Changes in light intensity have been demonstrated to directly affect the EPA content of *P. tricornutum*. When the light intensity was increased from 30 μmol photons m^−2^ s^−1^ to 180 μmol photons m^−2^ s^−1^, the EPA content of fatty acids was observed to decrease from 17.2 to 13.8% [75]. Moreover, an increase in light intensity from 150 μmol photons m^−2^s^−1^ to 750 μmol photons m^−2^ s^−1^ was accompanied by a decrease in EPA content, with a reduction of 5.73% [90]. The EPA biosynthesis pathway in *P. tricornutum* involves the transcription of six major gene products (four desaturation steps and two elongation steps) [90]. Of the four desaturase genes analyzed (*PTD5α*, *PTD5β*, *PTD6*, and *PTD15*), only *PTD5α* was significantly upregulated in response to high-intensity light exposure (750 μmol photons m^−2^s^−1^). Conversely, the transcription of the other desaturase genes was significantly reduced across all the treatments, which also explains the observed decrease in the EPA content of *P. tricornutum* under high light intensity. The wavelength of light also affects the fatty acid content; a shift from red to blue light increased the lipid content of *P. tricornutum*, while a shift from blue to red light promoted the synthesis of carbohydrates [76]. BL enhanced *P. tricornutum*’s capacity to produce fatty acids, while RL resulted in a notable elevation in the proportion of PUFAs [91], so light quality must be adjusted to increase PUFA production. The utilization of yellow and blue light was conducive to the accumulation of total lipids in *Thalassiosira pseudonana*, with the contents of PUFA and EPA reaching over 34% and 16%, respectively, under both light conditions [92]. Therefore, in the large-scale cultivation of microalgae for fatty acid production, specific combinations of light sources need to be tailored to the growth characteristics of microalgae to improve the efficiency of light energy utilization.

### 4.2. Fucoxanthin

**Figure 4:**
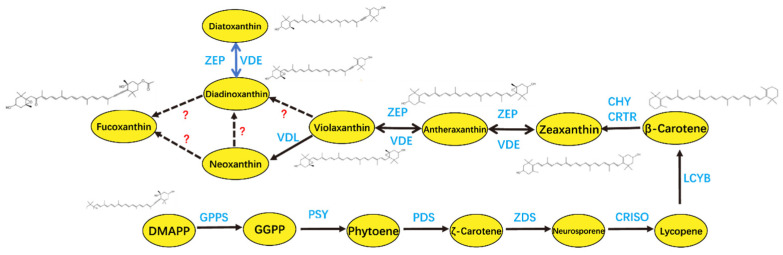
Fucoxanthin biosynthesis in *P. tricornutum* with recently investigated genes. DMAPP: dimethylallyl pyrophosphate; GPPS: geranylgeranyl pyrophosphate synthase; GGPP: geranylgeranyl pyrophosphate; PSY: phytoene synthase; PDS: phytoene desaturase; ZDS: ζ-carotene desaturase; CRISO: carotene isomerase; LCYB: lycopene B-cyclase; CHY: carotene hydroxylase; CRTR: carotene hydroxylase; VDE: violaxanthin de-epoxidase; ZEP: zeaxanthin epoxidase; VDL: violaxanthin de-epoxidase-like.

Fucoxanthin, a marine-derived xanthophyll carotenoid, is primarily localized in the chloroplasts of brown algae (e.g., kelp and wakame) and diatoms. It plays a dual role in light harvesting and photoprotection and has attracted attention for its bioactive properties in the nutraceutical and cosmetic industries [93,94]. A multitude of in vitro and in vivo studies have substantiated the multifaceted pharmacological effects of fucoxanthin, including anticancer, antidiabetic, anti-inflammatory, and antioxidant properties [95]. Fucoxanthin is also widely used as a food additive, commonly incorporated into functional food products as a natural antioxidant [96], and also exhibits antimicrobial activity, with the potential to extend the shelf life of plant-based dairy products when incorporated into plant-based dairy alternatives [97]. The biosynthetic pathway of fucoxanthin in *P. tricornutum* has been largely elucidated (Figure 4). The production of fucoxanthin in *P. tricornutum* is highly sensitive to environmental conditions [98], with light conditions identified as a key factor influencing the content of fucoxanthin in cells [77,99]. *P. tricornutum* produces more fucoxanthin in low-intensity light (20 μmol photons m^−2^ s^−1^) and 100 μmol photons m^−2^ s^−1^ conditions than in high-intensity light (200 μmol photons m^−2^ s^−1^) conditions [78]. The expression of several key genes involved in carotenoid synthesis (*PtPSY*, *PtPDS*, and *PtVDL1*) and the formation of the fucoxanthin–chlorophyll protein (FCP) complex (*PtLhcf5* and *PtLhcf8*) were identified by RNA sequencing and LC-MS/MS analysis in response to changes in light intensity [100]. A significant reduction in *PtPDS* gene expression was observed with increased light intensity, resulting in only one-tenth of the expression value under low light conditions. The expression of *PtLhcf5* and *PtLhcf8* exhibited a comparatively minor decline, amounting to approximately 20%. Light quality also influences the synthesis of fucoxanthin in *P. tricornutum*; the transition from white LED light to red LED light has been shown to enhance the expression of genes such as *PSY*, *ZEP*, *VDE*, and *FCP-b*, resulting in a notable increase in fucoxanthin accumulation [101]. In the presence of RL, the expression of genes associated with the light-harvesting protein complex was found to be upregulated, thus promoting the capture and conversion of light energy. Concurrently, fucoxanthin levels exhibited an analogous increase [102]. Under blue-green light conditions, the expression of genes involved in glycolysis, the tricarboxylic acid (TCA) cycle, and terpene skeleton biosynthesis is upregulated, thereby facilitating the synthesis of intracellular organic matter and promoting fucoxanthin accumulation. However, blue light is generally less effective than red light in enhancing fucoxanthin biosynthesis [103]. In the future, it will be necessary to optimize the ratio of red and blue LEDs to maximize the yield of fucoxanthin. A study employed LED lamps with adjustable spectral ratios as a tunable light source for the marine diatom *Odontella aurita*. When the red-light (RL)-to-blue-light (BL) ratio was set at 8:2, fucoxanthin productivity reached 9.41 mg L^−1^ d^−1^ [104]. These findings highlight the critical role of spectral optimization in maximizing the yield of high-value metabolites in microalgal biotechnology.

### 4.3. Microalgal Protein

Microalgae are characterized by elevated protein levels, in addition to a comprehensive array of amino acids that are indispensable for the human body. The amino acid values are comparable to those found in high-quality protein sources [105], including egg whites, lactoglobulin, and soybean [106]. A significant number of PB meat and seafood products that contain microalgae proteins have already been developed [10,107]. Microalgal proteins have minimal adverse effects on the nutritional value and flavor of plant foods. In addition, these proteins have excellent solubility, emulsification, gelation, and foaming properties, and therefore have a wide range of uses in the food industry [108]. The primary microalgal species used for protein production include *Spirulina* sp., *C. vulgaris*, and *Dunaliella salina*. *Spirulina* sp., a cyanobacterium rich in phycocyanin and essential amino acids, is widely recognized for its nutritional and medicinal value. Its high protein content and abundance of bioactive compounds makes it a valuable functional ingredient in human health foods, aquaculture feed, and livestock nutrition. Notably, it exhibits well-documented antioxidant and immunomodulatory properties [109,110]. Owing to its favorable safety profile and robust productivity, *Spirulina* sp. has been extensively studied as a sustainable alternative to the traditional protein sources in both food and feed industries. *Spirulina* sp. biomass is characterized by high carbohydrate and protein contents. *Spirulina* sp. is a blue-green bacterium, with phycocyanin being of particular significance due to its functional properties [111]. In one study, the maximum protein content (37%) in *Spirulina* sp. was achieved at the lowest light intensity (9 μmol photons m^−2^ s^−1^) [112].

Furthermore, the biomass growth and protein content of *Spirulina* sp. are influenced by the photoperiod [113]. The results indicated that the highest biomass concentration (0.51 g/L) and the maximum protein content (26.2%) were attained under a 14L:10D photoperiod. *Spirulina* sp. was also found to have a preference for different wavelengths of light; the highest dry cell weight of 0.343 g/L was recorded for a white light-emitting diode. In contrast, the highest protein content of 64.10 ± 0.44% was registered with a blue light-emitting diode [114]. *C. vulgaris* is a species of edible algae that is notable for its high protein content [115]. It has been demonstrated that protein content is less sensitive to fluctuations in light intensity [116]. Similar to *Spirulina* sp., the protein content of *C. vulgaris* under BL conditions was found to be higher than that under red or white light conditions [117]. Using ^14^C-labeled amino acids, some researchers observed that blue light increased the incorporation rate of labeled amino acids into proteins, suggesting enhanced protein synthesis efficiency under blue light conditions. In the context of algae-based protein production, a two-stage cultivation process can be employed, with the quality of light being a pivotal factor in achieving the desired outcome. A recent study has demonstrated that the production of biomass and protein in *D. salina* can be significantly enhanced through the regulation of light quality and the implementation of a two-stage cultivation method. In the initial phase, the utilization of a light source comprising dynamically enhanced WL is paramount. After accumulating a certain amount of biomass using blue LED or red LED light as a light source to continue cultivation, the second stage of this method led to a notable enhancement in protein production, with the utilization of either red or blue light proving to be particularly effective [118]. Algal protein synthesis exhibits relatively low sensitivity to fluctuations in light intensity. Achieving high protein yields depends on three fundamental strategies: preventing photoinhibition, providing adequate blue light (BL), and ensuring sufficient nitrogen availability. These conditions collectively enhance nitrogen assimilation and promote the activity of protein biosynthetic pathways, making them essential for optimizing microalgal cultivation for protein-rich biomass.

### 4.4. Astaxanthin

**Figure 5:**
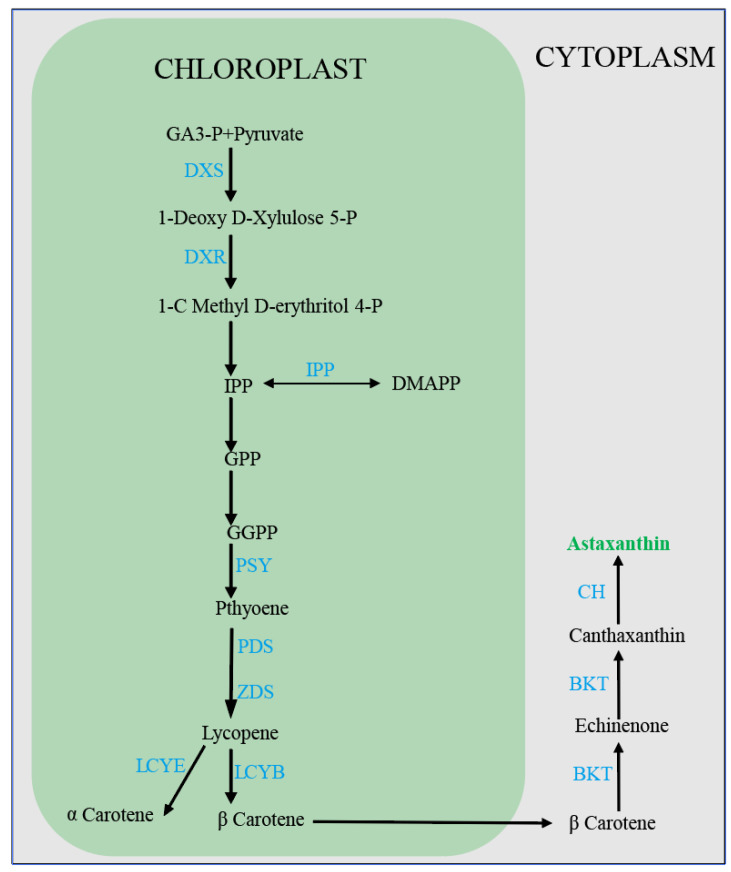
Synthesis process of astaxanthin in *H. pluvialis* cells. GA3-*p*: glyceraldehyde-3-phosphate; DXS: 1-deoxy-D-xylulose 5-phosphate synthase; DXR: 1-Deoxy- d-xylulose-5-phosphate reductoisomerase; IPP. isopentenyl pyrophosphate; DMAPP: dimethylallyl pyrophosphate; GPP: geranyl pyrophosphate; GGPP: geranylgeranyl pyrophosphate; PSY: phytoene synthase; PDS: phytoene desaturase; ZDS: ζ-carotene desaturase; LCYB: lycopene *β*-cyclase; LCYE: lycopene ε-cyclase; BKT: *β*-carotene ketolase; CH: -carotene hydroxylase; GPP: geranyl pyro phosphate BKT: *β*-carotene ketolase; CH: *β*-carotene hydroxylase.

**Figure 6:**
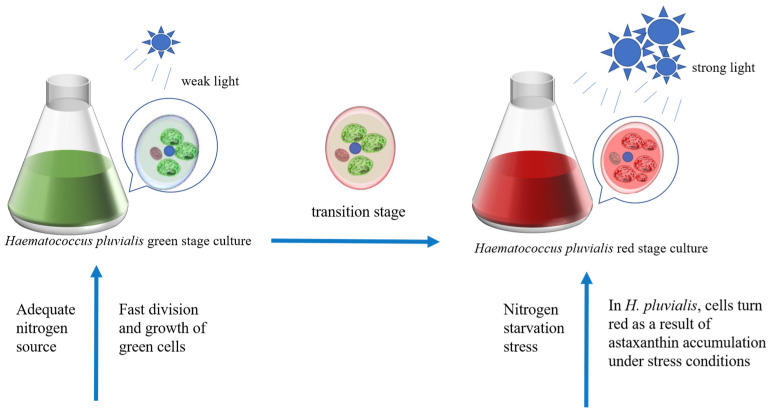
The two-step incubation method of *H. pluvialis*. The green phase provides sufficient nitrogen and suitable light conditions for rapid biomass accumulation, and the red phase promotes the accumulation of astaxanthin in the cells of *H. pluvialis* under the combined stress of nitrogen starvation and high-intensity light.

Astaxanthin (3,3′-dihydroxy-*β*, *β*-1-carotene-4,4′-dione) is a natural carotenoid [119] with superior antioxidant properties compared to those of other carotenoids and vitamin E [120]. Astaxanthin can provide vibrant colors to plant-based foods. In addition to this capacity, it has been shown to possess antioxidant, antineoplastic, and hypotensive properties when incorporated into functional foods [121,122]. *H. pluvialis* is one of the most important sources of natural astaxanthin, and the pathway of its biosynthesis has been fully elucidated (Figure 5). The two most critical factors affecting growth and astaxanthin synthesis in *H. pluvialis SAG 34-7* are light conditions and nitrogen concentration [123]. It is hypothesized that nitrogen starvation in combination with strong light is the optimal method for triggering the hyperaccumulation of astaxanthin in *H. pluvialis* (*strain H*_2_) [80,124]. However, one of the adverse effects of nitrogen starvation is a reduction in chlorophyll concentration within cells. Alterations in the concentration of photosynthetic pigments significantly influence the light absorption of *H. pluvialis*, which, in turn, affects the growth and reproduction rate of algal cells. The proposal of the two-step incubation method effectively addresses this issue [125] (Figure 6). The initial phase of the astaxanthin production process entails the cultivation of the green cells of *H. pluvialis* to accumulate a sufficient biomass. *H. pluvialis* exhibited optimal growth at a light intensity of 70 μmol photons m^−2^ s^−1^, which facilitated the expeditious accumulation of biomass [126,127]. However, in the red phase of *H. pluvialis*, intense light exceeding 100 μmol photons m^−2^ s^−1^ enhances natural astaxanthin accumulation [128]. Additionally, ROS generated as a consequence of photodamage act as a cellular signal to activate cellular metabolism in *H. pluvialis* [129], thereby promoting astaxanthin synthesis. At the gene transcription level, genes encoding specific enzymes required for *β*-carotene synthesis (*psy*,*pds*,*zds*,*crt-iso*,*lycB*) and related genes encoding key enzymes for bio-astaxanthin synthesis (*bkt* and *crtr-b*) exhibited increased expression upon increased light intensity [130,131,132]. Light saturation is uncommon in large-scale outdoor culture conditions, particularly during the red stage, which may be attributed to light scattering and mutual shading caused by high-cell-density cultures, which reduces the actual absorbed light irradiance. Therefore, higher astaxanthin accumulation in *H. pluvialis* can be achieved by combining nitrogen starvation with intense light in a large-scale culture. The cultivation of microalgae for astaxanthin production is also contingent upon the quality of light. In a particular study [79], astaxanthin production in *H. pluvialis* was observed to be most prolific under 3R4B (three red and four blue LED units) treatment, with a yield of 15.28 g/L. This yield was 2.2 times higher than that obtained under white light (WL) control conditions. In the context of future astaxanthin production in *H. pluvialis*, the use of combined red and blue light sources warrants consideration. This lighting strategy shows potential for enhancing the yield of a target compound.

### 4.5. Microalgal Polysaccharides

Plant-based meat substitutes are meat analogues that are typically characterized by their traditional and readily available nature, with their composition typically comprising plant proteins, polysaccharides, water, and oils [133]. Tissue-organized vegetable protein (TVP) is a structured vegetable protein that is produced through an extrusion process, the purpose of which is to mimic the fibrous structure of meat [134]. TVP can incorporate polysaccharides and non-textured proteins as a binder, thereby enhancing the water-holding capacity (WHC) and textural properties of PB meat analogues. Polysaccharides play a pivotal role as a suitable binder for the formation of stable gels [135]. Microalgal polysaccharides have been widely used in the food industry [136], and their incorporation has been shown to enhance the rheological characteristics of food substances, thereby contributing to their functionality and practical applications [137]. The utilization of PB foodstuffs fortified with microalgae polysaccharides has emerged as a novel approach to weight management. Research demonstrated that the administration of microalgae polysaccharides to mice with extreme obesity leads to the regulation of body weight and blood lipid levels, in addition to the restoration of beneficial intestinal bacteria [138]. Microalgal polysaccharides could play an important role in future PB foods, improving both their physical structure and nutritional value. Microalgal polysaccharide synthesis is also affected by light conditions. Frick et al. [139] observed that the highest chrysolaminarin content was attained under 50 μmol photons m^−2^ s^−1^ growth conditions of *P. tricornutum*. In addition to *P. tricornutum*, *β*-glucan was likewise produced mainly as chrysolaminarin in *Haptophyta* and *Heterokontophyta*. Light intensity was identified as a significant factor influencing the production of chrysolaminarin in these microalgae [140]. The transcript levels of genes were also compared between the light and dark cycles in *O. aurita*, and the expression levels of genes involved in the biosynthesis of chrysolaminarin, including *UGPase/PGM*, *UGP1-2*, *UGPT*, *BGS*, and *TGS1-3*, were observed to be higher at the beginning of the light period than in the dark period [141]. In *Isochrysis zhangjiangensis*, a marked elevation in the concentration of chrysolaminarin was discerned under high-intensity light in comparison to the corresponding cultures under low-intensity light, particularly on the initial day of incubation [142]. Similarly, Christian Schulze et al. [143] observed that the *β*-glucan content in *Spirulina* sp. platensis cells increased from 6.4% to 19.5% following an increase in light intensity from 50 μmol photons m^−2^ s^−1^ to 150 μmol photons m^−2^ s^−1^; however, when the light intensity was excessively high, there was a subsequent decrease in the content. Consequently, the light intensity should be regulated within a range of 200 μmol photons m^−2^ s^−1^ and adapted temporally by the concentration of algal cells.

## 5. Co-Regulatory Effects of Light and Other Environmental Factors

The physiological processes of microalgae are influenced by a combination of environmental factors. In addition to light conditions, the key variables include temperature; nitrogen availability; and the presence of exogenous additives, such as growth regulators and stress inducers [144,145]. Understanding the interactive effects of these factors is essential for optimizing microalgal cultivation systems aimed at maximizing biomass yield and target metabolite production. In general, the sensitivity of photosynthetic plants to light conditions is subject to changes in response to adjustments in ambient temperature [146], as do microalgae. There is evidence to suggest that the light saturation point of photosynthetic algae varies with temperature and decreases with a reduction in temperature [147]. A mathematical model has been developed to evaluate the photoautotrophic growth of *P. tricornutum* under a variety of incubation conditions, specifically regarding temperature and light intensity [148]. The optimum cultivation temperature and light intensity conditions were confirmed to be 21 °C and 496 μmol m^−2^s^−1^ at a constant aeration rate, respectively. It is noteworthy that a separate study demonstrated that the maximum biomass concentration, triacylglyceride (TAG) content, and EPA content of *P. tricornutum* under nitrogen starvation were each influenced by light intensity independently [149]. This indicates that light intensity and nitrogen content may be important interrelated factors affecting PUFA biosynthesis in *P. tricornutum*. The results of mass balance analyses demonstrated that under nitrogen starvation, *P. tricornutum* broke down some of the photosynthetic membrane lipids for the synthesis of EPA. However, the result is still a decrease in EPA content due to nitrogen starvation [150]; ensuring adequate nitrogen sources can therefore increase EPA yields. A study found that the activity of *H. pluvialis* cells dropped a lot in the first three days under strong light; lowering the nitrogen concentration in the culture can help restore their activity [151]. The results demonstrate that nitrogen starvation enhances the adaptive capacity of *H. pluvialis* to fluctuations in light intensity. The growth and astaxanthin accumulation of *H. pluvialis* are significantly influenced by the interaction between light intensity and nitrogen source concentration in a medium [152]. Nitrogen starvation is a prerequisite for the accumulation of astaxanthin in *H. pluvialis*; the effect of light factors on astaxanthin production in *H. pluvialis* needs to be investigated on this basis.

*H. pluvialis* can produce more astaxanthin during its red cell phase when affected by intense light, nitrogen deficiency, high salt conditions, or high temperatures. However, intense light also causes too many ROS to form, which can damage cells [153,154]. The incorporation of amino acids into the culture medium is anticipated to mitigate the level of ROS and enhance the biomass and astaxanthin production of *H. pluvialis* [155]. Previous research has demonstrated that the exogenous administration of arginine can augment the chlorophyll content and the expression of antioxidant activity genes in *H. pluvialis*, thereby reducing the detrimental effects of ROS [156]. Furthermore, in the process of culturing *Isochrysis* sp. for fucoxanthin production, it was observed that the incorporation of a low concentration of spermidine could stimulate algal cell proliferation and enhance fucoxanthin production. The addition of spermidine at a low light intensity of 20 μmol m^−2^ s^−1^ led to a maximal fucoxanthin yield of 6.11 mg/g [157]. In general, optimizing light conditions alone is insufficient to maximize yield and economic efficiency in microalgal production. To effectively regulate microalgal physiological activities and achieve synergistic improvements, other environmental factors must also be taken into account. These include factors such as temperature, pH, nutrient availability, and CO_2_ concentration, which collectively influence growth kinetics and metabolite accumulation.

## 6. Safety of Microalgae in Food Applications

The issue of food safety has always represented a primary concern for a variety of regulatory agencies. Consequently, any potential consumer hazards must be considered when promoting microalgae-based foods [158]. The safety of microalgal foods depends on the type of microalgae used. In the development and production of plant-based foods containing microalgae or microalgae derivatives, selecting the appropriate microalgae species following the existing food safety regulations is necessary. In Europe, the use of microalgae in food applications is strictly regulated under the Novel Foods Regulation (EU) 2015/2283 [159]. By 2024, only two species, *C. vulgaris* (a green microalga) and *Spirulina* sp. (a cyanobacterium commonly referred to as blue-green algae), will be authorized for human consumption. Under Novel Food legislation in Europe, the consumption of *Odontella aurita*, *Tetraselmis chuii*, and *Euglena gracilis* (a euglenozoan, but classified as microalgae for commercial purposes) is authorized. Other microalgal species are not permitted as food unless they undergo the novel food approval process, which requires comprehensive scientific safety assessments. The safety of microalgae is also contingent on the conditions of their culture and processing, which are frequently disregarded. These organisms can accumulate toxic compounds from various sources, including water and commercial fertilizers. In some cases, microalgae can adsorb toxic substances in water, including heavy metals, dyes, and antibiotics. Furthermore, open cultivation systems for microalgae present inherent contamination risks due to invasive microalgae, potentially jeopardizing product safety in scaled operations. To mitigate these safety risks, implementing a comprehensive water environment monitoring program is essential [160]. This initiative should focus on detecting emerging pollutants and harmful algal species, while establishing correlations between the microalgal product safety profiles and the cultivation/processing conditions. It is imperative to analyze the relationship between the toxicity/safety of microalgae products and the culture and processing conditions for the establishment of safe and efficient regulatory systems. Nethravathy MU et al. [161] pointed out some “checkpoints” that are very important for the safety of microalgae products: (1) the open environment must be regularly checked for biological and non-biological pollutants, (2) water quality must be checked, (3) potential health problems caused by a high nucleic acid content must be assessed, (4) the potential allergic risks of microalgae must be assessed, and (5) the safety of the entire production process must be assessed from upstream to downstream. The safe application of microalgae in the food industry can be ensured through rigorous safety testing in the selection of algal species, water quality control, biomass harvesting, and product extraction. In the future, the large-scale cultivation of microalgae for PB food production will require strict compliance with the existing laws and regulations, alongside relevant scientific literature. This is essential to ensure the safety of cultivation practices, the proper utilization of microalgal biomass, and the overall legality and acceptability of microalgae-derived food products. As the regulatory landscape continues to evolve, interdisciplinary collaboration among researchers, policy makers, and industry stakeholders will be critical to the safe and sustainable integration of microalgae into the human food chain.

## 7. Prospects

### 7.1. Application of AI in Microalgae Cultivation

**Figure 7:**
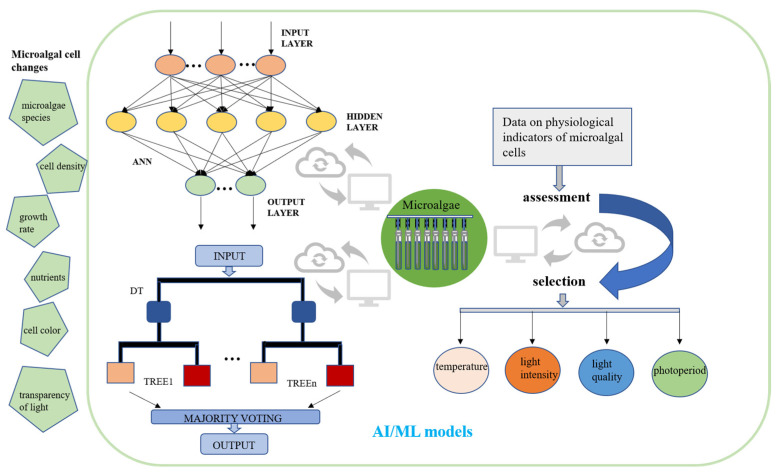
A conceptual framework for integrating artificial intelligence and machine learning in optimizing large-scale microalgae cultivation systems. Using artificial intelligence to analyze the growth of microalgae cells and physiological indicators, such as microalgae growth rate and cell density, and then regulate the temperature and light conditions. AI, artificial intelligence; ML, machine learning; ANN, artificial neural network; DT, decision tree.

Artificially illuminated plant factory technology has matured in recent years. The integration of artificial intelligence with LED lighting has proven to offer several key benefits. First, these facilities exhibit high levels of resource utilization, resulting in enhanced productivity per unit area of land. Secondly, they demonstrate resilience to weather conditions, thereby ensuring a consistent performance regardless of external factors [162]. AI is also becoming more popular in the study of microalgae because AI algorithms can provide useful information in situations with a lot of uncertainty. In general, AI algorithms can be divided into three types: machine learning, metaheuristics, and expert systems [163]. Before using AI to detect the microalgae growth process and dynamically adjust real-time culture parameters, a substantial volume of extant data concerning the growth of microalgae influenced by environmental factors must be entered into the AI system. This data is essential for training the AI to develop an accurate predictive model [164]. AI can analyze large amounts of data collected by sensors, which include temperature, humidity, carbon dioxide concentration, pH, and nitrogen and phosphorus concentrations [165]. These data can be used to learn about the growth of microalgae and the build-up of target products. In addition to the analysis of environmental factors, it is imperative to analyze the microalgal biomass concentration, photosynthetic activity, and cell color in conjunction with hardware facilities [166]. Subsequently, the decision information is analyzed and evaluated by the artificial neural network and the decision tree, and the computer then regulates the temperature, humidity, light intensity, and light quality of the control port of the microalgae cell factory (Figure 7). Currently, only one kind of lamp is usually used to provide the light for algal cell photosynthesis in microalgae mass cultures. However, the growth process of algal cells is dynamic, and fixed light conditions cannot meet the needs of algal cells at the different stages of development. The utilization of AI for the analysis of microalgae growth, with the subsequent adjustment of light intensity and the employment of LED lamps of varying colors to provide microalgae with different light qualities are indicative of a broad range of potential applications. Optimal illumination can effectively enhance the accumulation of microalgal biomass and the production of valuable products. With recent advancements in artificial intelligence, the integration of AI-driven technologies capable of analyzing the algal growth dynamics is being explored. The subsequent real-time adjustment of environmental parameters, such as light intensity, temperature, and humidity, holds considerable potential for enhancing the efficiency and sustainability of microalgae cultivation. Integrating AI-based solutions promises to reduce labor requirements, material consumption, and optimize the operational efficiency of microalgae production. More significantly, AI-regulated microalgae cell factories hold strong potential as a major future source of PB food. Their inherent intelligence and capacity to minimize contamination risks directly address critical food safety concerns.

### 7.2. Limitations and Solutions

Microalgae are seen as a valuable source of nutrients for new PB foods. Microalgae biomass is rich in essential nutrients needed for good health, along with some active ingredients that offer health benefits. These factors will be a key part of the development of plant-based foods in the future. Despite recent advancements in research on breeding and cultivating high-value microalgae, the large-scale culture of microalgae and the harvesting of biomass for subsequent industrial production remain in their infancy. The selection of suitable and cost-effective light solutions for the growth of microalgae in large-scale cultivation is of paramount importance for the expansion of the microalgae industry. The current state of research on the impact of light conditions on the growth and productivity regulation of microalgae is characterized by the following limitations:

1. The research methods employed to study the impact of light on microalgae growth and productivity exhibit considerable inconsistency, resulting in a notable decline in the reliability of experimental outcomes. To ensure consistency, it is essential to provide comprehensive information in experimental studies, such as details on the light parameters, the microalgae concentration, the medium volume, the composition, as well as the concentration and the flow rate of CO_2_.

2. Conventionally, the cultivation of microalgae is predominantly characterized by constant light intensity and wavelength. However, in the future, it is imperative to formulate dynamic light intensity and wavelength changes, taking into account the unique characteristics of various microalgae species. This approach is more in line with the dynamic process of microalgae growth. To solve these problems, it is first necessary to accumulate a large amount of data from a large number of microalgae cultivation experiments. This data must then be analyzed through artificial intelligence, and the results must be used to train an AI model to determine the optimal environmental factors required during the growth of a certain type of microalgae. It should be noted that these factors are subject to change.

## 8. Conclusions

Microalgae are widely recognized as a vital component of plant-based (PB) foods and a sustainable source of diverse bioactive compounds. Microalgal cells are rich in high-quality proteins, essential amino acids, polyunsaturated fatty acids, and natural pigments, which can effectively compensate for the nutritional deficiencies commonly associated with the traditional PB foods. By precisely regulating cultivation conditions, specific functional components in microalgae can be enriched, allowing for targeted nutritional enhancement. Due to their highly efficient photosynthetic autotrophy, the biomass yield of microalgae per unit of land area can exceed that of conventional crops by more than tenfold. Moreover, their growth requires only sunlight, water, and carbon dioxide, significantly reducing reliance on freshwater and arable land. Currently, microalgae have been successfully applied in plant-based meat texture enhancement, dairy alternatives, and functional food additives. Natural pigments extracted from microalgae also serve as sustainable replacements for synthetic colorants in food formulations. Light is a fundamental requirement for the photoautotrophic growth of microalgae. The key parameters, such as light intensity, spectral composition, and the photoperiod, serve as effective tools for regulating microalgal growth, metabolic pathways, and the biosynthesis of biochemically valuable compounds. The optimal light intensity for most microalgal species typically ranges from 50 to 400 μmol photons m^−2^ s^−1^. However, a few species can tolerate and grow under intensities exceeding 3000 μmol photons m^−2^ s^−1^ due to their strong photo-regulatory capabilities. Variations in light intensity and wavelength can significantly influence lipid accumulation, fatty acid profiles, pigment synthesis, as well as protein and polysaccharide contents. Accordingly, light conditions can be dynamically adjusted, either in response to real-time growth status or tailored to the biosynthetic characteristics of target compounds, to improve light energy utilization efficiency.

To advance the development of microalgal cell factories, smart light regulation within a plant factory framework is essential for optimizing light energy utilization and enhancing biomass productivity. Artificial intelligence (AI) models trained on cultivation data can dynamically adjust light intensity according to specific growth stages, while programmable LED systems can synergistically accelerate microalgal growth and improve energy efficiency. Integrating such intelligent lighting strategies with environmental sensors and metabolic models may enable fully autonomous and energy-efficient algal cultivation systems in the future.

## Figures and Tables

**Table 1:** Some international companies that use natural or artificial light sources for mass cultures and their main products. (Tabulated data were compiled from official corporate portals and annual reports of respective entities.)

Company Name	Light Source Used	Species of Microalgae Cultured	Major Products
Arizona Algae Products LLC (USA)	Solar light	*Spirulina* sp., *Chlorella vulgaris*	Microalgal oil and powder
Vaxa Technologies(IS)	Solar light	*Cyanobacteria*	Phycocyanin biofuels and algal proteins
BioFields (MX)	Solar light	*Spirulina* sp., *Chlorella vulgaris*	Aquatic feed
Susewi (MOR)	Solar light	*Brown algae, Green algae*	Diatom material Algica, biofertilizer
Swedish Algae Factory (SE)	Solar light	*Diatoms*	Sustainable aviation fuels, microalgal proteins, cosmetics, and pharmaceuticals
Chitose Carbon Capture Central (MAS)	Solar light	*Diatoms, Spirulina* sp., *Chlorella vulgaris*	Natural pigments, cosmetics, and animal feed additives
Provectus Algae (AUS)	Artificial light	*Chlorella vulgaris, Phaeodactylum tricornutum, Dunaliella salina*	Natural astaxanthin, health products, cosmetics
Yunnan Xi Zao Biotechnology (CHN)	Artificial light	*Haematococcus pluvialis*	Biofuels, food additives
Festo (GER)	Artificial light	*Cyanobacteria*	Pharmaceutical, cosmetic, and bioplastic
Provectus Algae (USA)	Artificial light	*Asparagopsis, Dunaliella salina, Diatoms*	Natural pigments, health products, and cosmetics

**Table 2:** Optimum light intensity values for maximum growth rates of algae of different taxonomic groups.

Investigated Light Intensity (μmol Photons m^−2^ s^−1^)	Optimal Light Intensity (μmol Photons m^−2^ s^−1^)	Microalgae Species	Marine or Freshwater Algae	Reference
60, 100, 250, 500, 750	60–112	*Phaeodactylum tricornutum*	marine algae	[41]
200, 500, 1000, 1500, 2000	1500	*Dunaliella salina*	marine algae	[42]
70, 140, 210	70	*Porphyridium purpureum*	marine algae	[43]
50, 125, 325	325	*Isochrysis galbana*	marine algae	[44]
20–500	300	*Arthrospira fusiformis*	marine algae	[45]
60, 195, 330, 465, 600	110–220	*Rhodomonas* sp.	marine algae	[46]
75, 100, 150, 500, 660, 750	660	*Synechococcus* sp.	marine algae	[47]
5, 25, 50, 100, 250, 850	26–55	*Microchloropsis salina (=Nannochloropsis salina)*	marine algae	[48]
35, 200, 400	400	*Lobosphaera incisa*	freshwater algae	[49]
150, 300	150	*Chromochloris zofingiensis (=Chlorella vulgariszofingiensis)*	freshwater algae	[50]
50, 150, 300, 500	150	*Chlorella vulgarisvulgaris*	freshwater algae	[51]
50~300	150	*Haematococcus pluvialis*	freshwater algae	[52]
50, 150, 300	150	*Scenedesmus obliquus*	freshwater algae	[53]
10, 50, 150, 200, 350, 1000	150	*Tetradesmus obliquus*	freshwater algae	[54]

**Table 3:** Microalgal biomass and microalgal extracts in plant-based foods.

Microalgae Products for Plant-Based Foods	Descriptions	Microalgae Species	Reference
Microalgae protein	Plant-based meat and plant-based milk	*Spirulina* sp., *Chlorella vulgaris*	[71]
Nutrient enhancer	Pigments, Antioxidant, Omega-3 Phycobiliproteins, Polysaccharides	*Haematococcus pluvialis*, *Scenedesmus obliquus*	[72]
Antioxidants	Improved food stability	*Tetraselmis* sp., *Dunaliella salina*, *Phaeodactylum tricornutumScenedesmus obliquus*	[73]
Food Flavourings	Flavor components of plant-based seafood alternatives	*Rhodomonas salina*, *Tetraselmis chui*, *Phaeodactylum tricornutum*	[74]

**Table 4:** Influence of light intensity and light quality on nutrient productivity of microalgae.

Species and Strain	Optimal Light Intensity and Quality (μmol Photons m^−2^ s^−1^)	Nutrients	Productivity	Reference
*Phaeodactylum tricornutum*	30 μmol photons m^−2^ s^−1^, blue light	EPA	Increase by 30% (17% of fatty acids)	[75,76]
	100 μmol photons m^−2^ s^−1^, red and blue (50:50) light	Fucoxanthin	Increase more than 100% (9~12 mg/g)	[77,78]
*Chlorella vulgaris*	100 μmol photons m^−2^ s^−1^, blue light	Protein	Increase by 35% (460 mg/g)	[58,62]
*Haematococcus* *pluvialis*	400 μmol photons m^−2^ s^−1^, red and blue (40:60) light	Astaxanthin	Increase by more than 100% (15.28 mg/L)	[79,80]

## Data Availability

No new data were created or analyzed in this study. Data sharing is not applicable to this article.
